# Holistic approaches in systemic lupus erythematosus: do physicians avoid addressing difficult-to-treat but highly relevant symptoms?

**DOI:** 10.1136/rmdopen-2024-005400

**Published:** 2025-03-07

**Authors:** Ioannis Parodis, Chris Wincup, Zahi Touma, Jeanette Andersen, Vibeke Strand, Christopher Sjöwall

**Affiliations:** 1 Division of Rheumatology, Department of Medicine Solna, Karolinska Institutet, Karolinska University Hospital, and Center for Molecular Medicine (CMM), Stockholm, Sweden; 2 Department of Rheumatology, Faculty of Medicine and Health, Örebro University, Örebro, Sweden; 3 Department of Clinical and Academic Rheumatology, King’s College Hospital, London, UK; 4 Division of Rheumatology, Toronto Lupus Program, University Health Network, Schroeder Arthritis Institute, University of Toronto, Toronto, Ontario, Canada; 5 Lupus Europe, Brussels, Belgium; 6 Division of Immunology/Rheumatology, Stanford University, Stanford, California, USA; 7 Department of Biomedical and Clinical Sciences, Division of Inflammation and Infection/Rheumatology, Linköping University, Linköping, Sweden

**Keywords:** Lupus Erythematosus, Systemic, Health-Related Quality Of Life, Patient Reported Outcome Measures, Treatment

## Abstract

Despite advancements in the management of systemic lupus erythematosus (SLE), patients experience poor health-related quality of life (hrQoL) and premature death due to disease severity and treatment side effects. Achieving remission offers substantial benefits, including improved hrQoL and reduced mortality, yet the complexity of SLE, with its diverse underlying immune mechanisms and clinical manifestations, hampers progress. Involvement of the central nervous system with symptoms like fatigue, pain and brain fog often goes unaddressed due to limited evidence-based guidance and measurement tools. This neglect reflects gaps in training, discomfort in addressing untreatable symptoms and an overemphasis on evidence-based medicine, compromising holistic care. Recognising patient-reported outcomes has shifted SLE care towards a more patient-centred model, addressing hrQoL and aligning treatment goals. Embracing this approach and prioritising symptom management, even when a definitive cure is lacking, ensures compassionate, comprehensive care that improves adherence, satisfaction and the overall lived experience of patients with SLE.

 Despite significant advancements in the management of systemic lupus erythematosus (SLE), patients continue to report poor health-related quality of life (hrQoL)^1 2^ and face an elevated risk of premature mortality, both from the disease itself and from the immunosuppressive treatments required to control it.^3^,^7^ While evidence-based medicine (EBM) has transformed the treatment of other conditions, such as rheumatoid arthritis,^8^ patients with SLE still await similar breakthroughs. Emerging data suggest that achieving ‘remission’, or at least a low disease activity state when remission is not possible, is critically important for patients,^9^ offering substantial benefits including reduced long-term damage,^10 11^ increased survival,^10 11^ improved hrQoL^12^ and reversal of dysregulation within critical pathways in SLE pathogenesis.^13^

The complexity of SLE,^14^ with its multiple underlying immune mechanisms,^15 16^ results in a range of manifestations that are often poorly understood. This complexity, among other things, contributes to the fact that many patients report hrQoL scores comparable to those of individuals with advanced cancer.^17 18^ One challenging aspect is the involvement of the central nervous system (CNS), where symptoms are frequently observed but vary widely, making it difficult to attribute them solely to SLE.^19^,^25^ CNS manifestations, focal and diffuse, may directly result from SLE disease activity, accrued damage or comorbid conditions. These symptoms may additionally be related to an intercurrent mental health diagnosis, which may be reactive to the underlying diagnosis of SLE (such as health-related anxiety or depression) or due to various psychosocial factors associated with a chronic illness. Yet, for patients who manage to maintain a low disease activity state over time, hrQoL outcomes show marked improvement in both the short and long term.^12 26 27^

Despite the debilitating nature of symptoms like fatigue, pain, brain fog and reduced quality of life, many of these symptoms go unaddressed in clinical consultations, often because of the lack of appropriate measurement tools for clinical settings or because of the limited evidence-based guidance for their treatment.^28 29^ Fatigue, for example, is a commonly reported symptom in SLE, yet it is frequently disregarded by clinicians,^1^ partly because there are few specific medications^30^ or non-pharmacological strategies^31^,^33^ to address it directly. This challenge is also apparent for rare but severe manifestations of active SLE, where the scarcity of high-quality clinical trials or observational studies limits evidence-based treatment strategies.^34^,^36^ Physicians may unconsciously avoid discussing these difficult-to-treat symptoms, focusing instead on manifestations with more established treatment pathways.^9^ This tendency, though rooted in the principles of EBM, falls short of a holistic approach that truly considers the full spectrum of patient experience.

This selective attention may, in part, reflect the strong emphasis on EBM within medical training today. It might also indicate an underlying discomfort with discussing symptoms that lack immediate solutions based on robust scientific evidence. Furthermore, many clinicians involved in the care of patients with SLE may lack training in identifying and managing these symptoms. A paucity of training in the identification of mental health symptoms and a lack of confidence in the treatment of these symptoms once identified may also be a key barrier to holistic care.^37^ Nevertheless, these aspects of patient experience are crucial, and asking about them, even in the absence of definitive treatments, is essential for holistic care. Recognising the significance of symptoms like fatigue and pain as part of the patient’s lived reality is essential to comprehensive SLE management. Achieving this recognition would require collaboration among multiple healthcare professionals, a need that demands both time and resources, factors that are often de-prioritised in current healthcare systems.

Increasing awareness of the importance of patient-reported outcomes (PROs) and PRO measures is changing the landscape of SLE treatment.^38 39^ Not only do PROs provide insights into how patients experience their illness, but their associations with short- and long-term disease outcomes also highlight their clinical relevance.^40 41^ Moreover, newer, more potent therapies have shown promise in improving PROs,^42^,^46^ as has the achievement of treatment goals such as remission and low disease activity.^12^ This growing focus on PROs is driving a more patient-centred approach in SLE care, with greater attention given to hrQoL concerns raised by patients themselves.

In reflecting on the Hippocratic Oath, we must remember that if we cannot cure, we must still aim to treat; and if we cannot treat, we should seek to relieve symptoms or console. Acknowledging and respecting symptoms for which there is no definitive treatment and managing them with compassion and the best available knowledge are critical aspects of comprehensive care. Fatigue, for instance, may sometimes have a treatable cause, such as hypothyroidism^47^ or depression,^28^ warranting investigation. Thus, an effective approach could involve first investigating potential underlying causes of reported symptoms and, after ruling out attribution to disease activity and the need for pharmacological management, directing attention to non-pharmacological interventions such as tailored physical activity programmes, patient education to enhance self-efficacy and psychosocial support.^33^ This recognition of symptoms that matter to patients promotes alignment between patients and their healthcare team, enhancing satisfaction and ultimately encouraging better adherence to therapy and collaboration with the healthcare team.^48^ By prioritising a more patient-centred approach, SLE care can evolve to address not only the science but also the lived experience of the disease, fostering a truly holistic model of care.
